# Supplementary material for: Abnormal phase transition between two-dimensional high-density liquid crystal and low-density crystalline solid phases
Source: Nat Commun. 2018 Jan 15;9:198. doi: 10.1038/s41467-017-02634-6 (PMC5768887; doi:10.1038/s41467-017-02634-6)
Supplement: Supplementary file 1 — Supplementary Information [file 41467_2017_2634_MOESM1_ESM.pdf]

Supplementary Note 1: CO on Cu(111) with coverage lower than 1/3 ML.

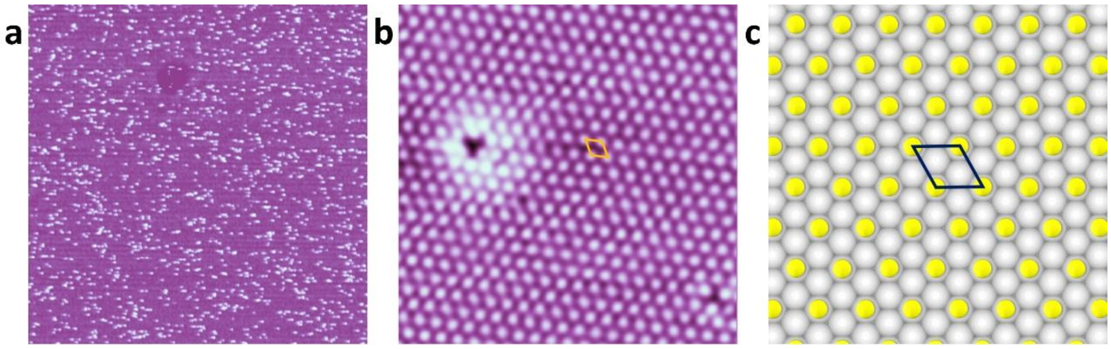

Supplementary Figure 1. CO on Cu(111) at low coverage. (a) STM image of CO on Cu(111) with coverage lower than 1/3 ML. The image is obtained at tip bias 200 mV and set point 196 pA; (b) STM image of (√3 × √3) R30° CO lattice on Cu(111) which is formed when the coverage reach 1/3. The tip bias is -1.0 V and set point is 497 pA. (c) The structural model of (√3 × √3) R30° superstructure of CO on Cu(111). The big silver and small yellow balls represent the Cu atoms and CO molecules, respectively. The rhombuses in (b) and (c) represent the unit cell of (√3 × √3) R30 ° superstructure. The scanning ranges are: (a) 28.0 nm × 28.0 nm; (b) 10.6 nm × 10.6 nm.

When the coverage of CO on Cu(111) with substrate temperature about 77 K is lower than 1/3 ML, we can't obtain the regular structure on Cu(111) in STM images. On the contrary, only irregular spikes are observed in STM image as shown in Supplementary Fig. 1(a). It is because that the CO molecules diffuse fast on the surface at 77K, and the tunneling junction between tip and surface is influenced during scanning. On the other hand, when the coverage of CO reaches 1/3 ML, a (√3 × √3) R30° superstructure with respect to Cu(111) is formed as shown in Supplementary Fig. 1(b), in which each bright protrusion represent on CO molecules on top of Cu atoms. The atomic structure of this (√3 × √3) R30° superstructure is shown in Supplementary Fig. 1(c), which has been reported by previous studies[1,2].

Supplementary Note 2: Progress of transition between α and β phases.

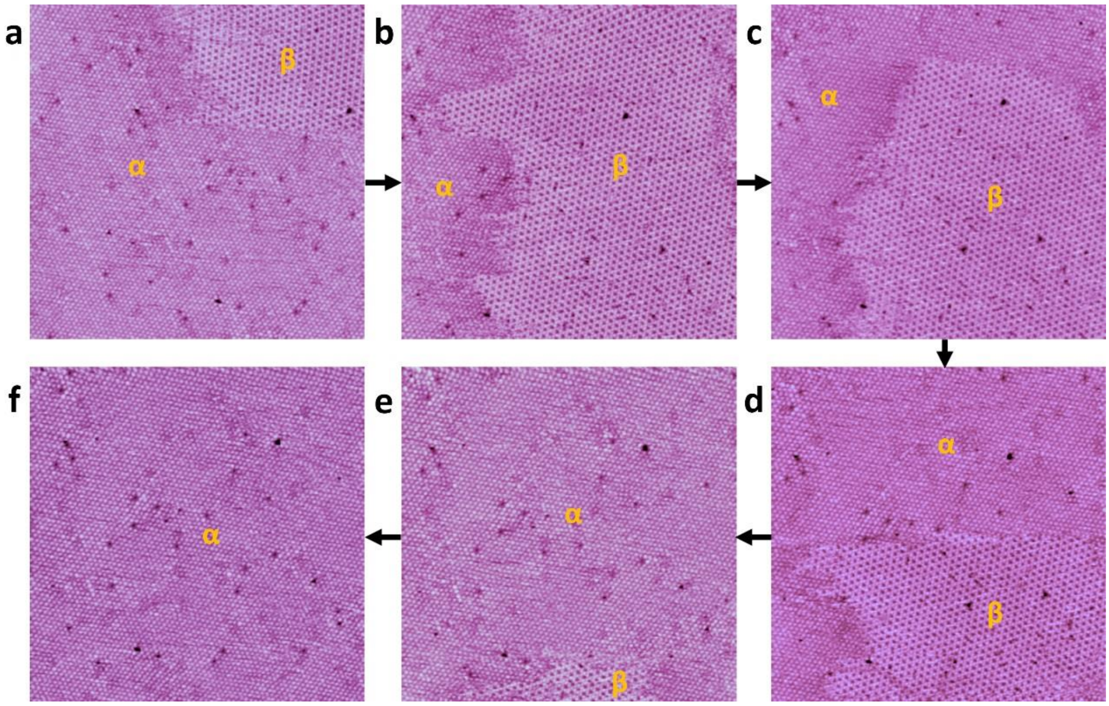

Supplementary Figure 2. Transformation process of α and β phases. (a-f) STM images were successively taken on same area with coexisting α and β phases of CO on Cu(111). The scan bias and set point are same (tip bias -2.00 V, set point 32 pA). The taking order is marked by arrows. The scanning size of the area is 60.0 nm × 60.0 nm.

The α and β phases can transform to each other stimulated by tip bias, and one example is shown by several successive STM images in Supplementary Fig. 2. Firstly, Supplementary Fig. 2(a) indicates the β phase just locates in the top right of image and other area is covered by α phase. After finished the image and scanning the same area again, we found that most area is covered by β phase, as shown in Supplementary Fig. 2(b). During the next several scanning cycles, the area of β phase gradually is shrinked (Supplementary Fig. 2(c-e)) and disappeared (Supplementary Fig. 2(f)). The transition progress reveals that α and β phase can transform to each other by tip bias, which indicates these two phases have similar stability and their CO densities are same. The detailed investigation for the reversible phase transition between α and β phases was also reported by B. Wortmann *et al.*[3].

Supplementary Note 3: The atomic structures of boundaries in γ phase.

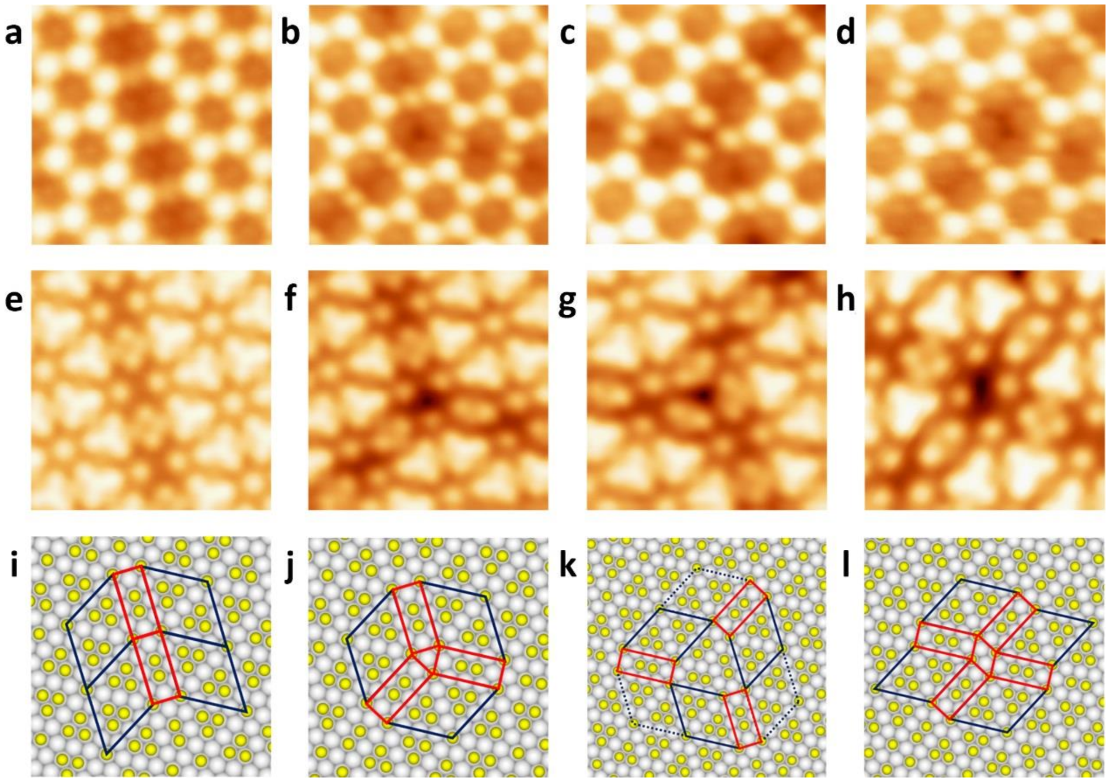

Supplementary Figure 3. Domain boundaries of γ phase. (a-d) High resolution STM images of different boundaries in γ phase of CO on Cu(111) taken by normal tungsten STM tip, respectively. (e-h) High resolution STM images of similar boundaries in γ phase as (a-d) taken by CO-decorated STM tip, respectively. All the STM images are obtained at tip bias -1.0 V and set point 186 pA. (i–l) The corresponding structural models of boundaries in γ phase shown in (a–d) or (e-h), respectively. The red rectangles and blue rhombuses represent the unit cells at boundaries and main part of γ phase, respectively. The big silver and small yellow balls represent the Cu atoms and CO molecules, respectively. The scanning sizes are: (a) 4.2 nm × 4.2 nm; (b) 4.2 nm × 4.2 nm; (c) 3.7 nm × 3.7nm; (d) 3.7 nm × 3.7 nm; (e) 2.9 nm × 2.9 nm; (f) 3.0 nm × 3.0 nm; (g) 2.8 nm × 2.8 nm; (h) 2.5 nm × 2.5 nm;

The γ phase of 2D CO on Cu(111) was observed at substrate temperature about 5K. From STM images (Fig.2(a) in main text), the γ phase consists of domains with perfect honeycomb lattice, which are separated by domain boundaries (DBs). We summary all kinds of DB s in Supplementary Fig. 3(a-d): the normal line-like DBs (Supplementary Fig. 3(a)), three line-like DBs meet at one point (Supplementary Fig. 3(b, c)), and four line-like DBs meet at one point. By attaching a CO molecule on the apex of tip, the

resolution of STM images (Supplementary Fig. 3(e-h)) is enhanced, from which we can found the DBs are mainly consisted of titled CO dimers and monomers. The structural model shown in Supplementary Fig. 3(i-l) reveal that the unit of DB is a rectangle and consists of a CO dimer at the center and a monomer at the corner.

Supplementary Note 4: The calculations of isolated CO clusters on Cu(111).

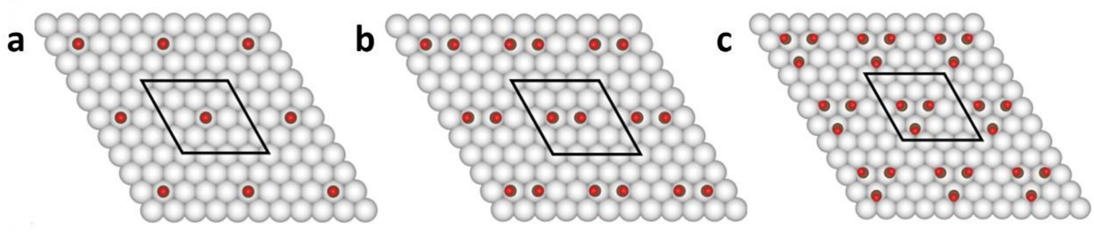

Supplementary Figure 4. Calculations of isolated CO clusters. The top views of optimized configurations for isolated CO (a) monomer, (b) dimer, and (c) trimer clusters on Cu(111) using DFT calculations. The black parallelograms represent periodicities of these cluster models.

The adsorption geometries of isolated CO clusters, including CO monomer, dimer, trimer, tetramer, and hexamer, are relaxed on adjacent top sites of Cu(111) using DFT. It is found that the isolated CO monomer, dimer, and trimer clusters are stabilized by the binding interaction with Cu(111), where the CO molecules occupy adjacent top sites, as shown in Supplementary Fig. 4. However, the single hexamer and tetramer of CO molecules cannot be maintained due to the large lateral repulsion.

Supplementary Note 5: The calculations on diffusion of CO on Cu(111).

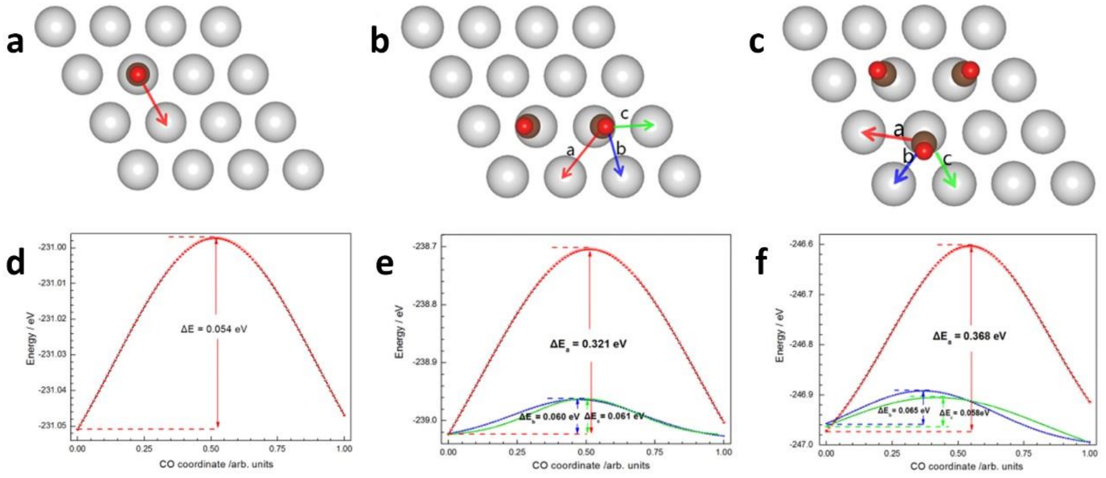

Supplementary Figure 5. Calculations on diffusion of CO. The structure model and corresponding calculated diffusion barrier for CO (a, b) monomer, (c, d) dimer, and (e, f) trimer on Cu (111) surface. Red, brown and grey balls in structural model represent oxygen, carbon and copper atoms, respectively. Red, blue and green arrows indicate diffusion direction of the CO molecule, corresponding to the red, blue and green profiles in (b), (d), (f).

The diffusion barrier on Cu(111) is important to understand the interactions between CO molecule and the surface, as well as intermolecular interactions. The calculated diffusion barriers of CO in monomer, dimer, and trimers are illustrated in Supplementary Fig. 5. It is found that the surface mobility of CO in dimer and trimer is anisotropic due to the intermolecular repulsion, however, the lowest diffusion barriers are comparable (~0.06 eV). The value of diffusion barrier is comparable to hydrogen bonding interaction, indicating very low diffusion rate of CO molecule on Cu(111) at low temperature. On the other hand, such moderate diffusion barrier also demonstrates the adsorption pattern of high dense CO on Cu(111) can be easily tuned by STM tip or thermal fluctuation.

Supplementary Note 6: Continuous movement of CO in α/β phases.

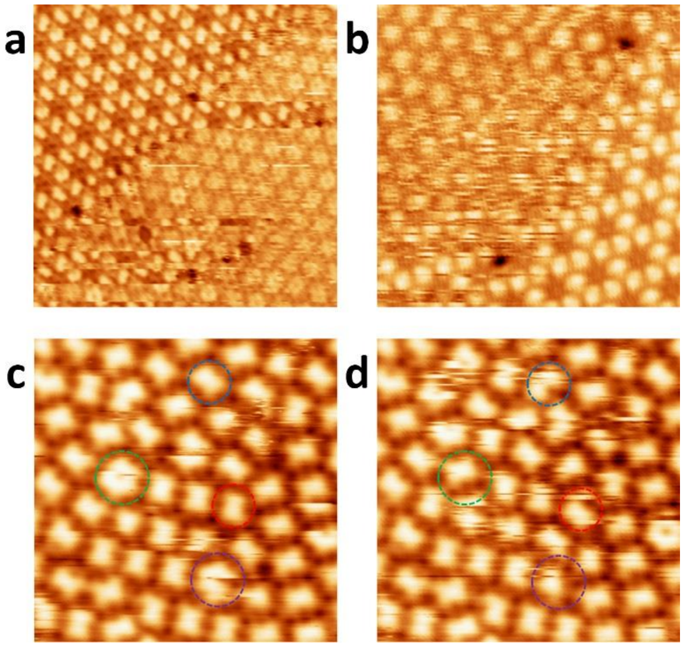

Supplementary Figure 6. Continuous movement of CO in α/β phases. (a, b) STM images taken on the area with boundaries separating α and β phases. (c, d) High resolution STM images of α phase in the same area but taken at different time. Examples of clusters transformation are marked by colors cycles. The scanning sizes of the images are: (a) 12.0 nm × 12.0 nm; (b) 10.0 nm × 10.0 nm; (c, d) 5.8 nm × 5.8 nm. The scanning parameters are: (a) V_{tip} = -500 mV, I = 301 pA; (b) V_{tip} = -477 mV, I = 195 pA; (c, d) V_{tip} = -1.00 V, I = 207 pA.

In our experiment, we can find that CO molecules at boundaries between α and β phases are moving all the time, as shown in Supplementary Fig. 6(a, b). In α phase, the CO clusters are also changing and moving, as shown in Supplementary Fig. 6 (c) and (d), which were obtained at different time on the same area. For examples, the CO clusters marked by cycles are transformed during scanning.

Supplementary References

1. Pritchard, J. On the structure of CO adlayers on Cu(100) and Cu(111). *Surf. Sci.* **79**, 231-244 (1979).
2. Bartels, L., Meyer, G. & Rieder, K. H. The evolution of CO adsorption on Cu(111) as studied with bare and CO-functionalized scanning tunneling tips. *Surf. Sci.* **432**, L621-L626 (1999).
3. Wortmann, B. *et al.* Reversible 2D phase transition driven by an electric field: visualization and control on the atomic scale. *Nano Lett.* **16**, 528-533 (2016).
